# Information and communication technologies for approaching smokers: a descriptive study in primary healthcare

**DOI:** 10.1186/1471-2458-15-2

**Published:** 2015-02-13

**Authors:** Elisa Puigdomènech, Jose-Manuel Trujillo-Gómez, Carlos Martín-Cantera, Laura Díaz-Gete, Mónica Manzano-Montero, Jessica Sánchez-Fondevila, Yolanda Gonzalez-Fernandez, Beatriz Garcia-Rueda, Elena-Mercedes Briones-Carrió, Mª-Lourdes Clemente-Jiménez, Carmen Castaño, Joan Birulés-Muntané, Grupo Estudio TABATIC

**Affiliations:** Unidad de Soporte a la Investigación Barcelona Ciudad, Instituto Universitario de Investigación en Atención Primaria Jordi Gol (IDIAP Jordi Gol), C/ Sardenya, 375, entresol, 08025 Barcelona, Spain; Centre d’ Atenció Primaria Passeig de Sant Joan, Institut Català de la Salut, Barcelona, Spain; Departament of Medicine, Universitat Autònoma de Barcelona, Barcelona, Spain; Centre d’Atenció Primaria La Sagrera, Institut Català de la Salut, Barcelona, Spain; Centre d’Atenció Primaria Horta 7F, Institut Català de la Salut, Barcelona, Spain; Centre d’Atenció Primaria Navarcles, Barcelona, Spain; Centro Sanitario Santo Grial (Huesca), Grupo Aragonés de Investigación en Atención Primaria, Asociación para la Prevención del Tabaquismo en Aragón (APTA), Aragón, Spain; La Alamedilla Health Centre, Castilla y León, Health Service–SACYL, redIAPP, IBSAL, Salamanca, Spain

**Keywords:** Smoking cessation, Information and communication technologies, Primary health care

## Abstract

**Background:**

Common interventions for smoking cessation are based on medical advice and pharmacological aid. Information and communication technologies may be helpful as interventions by themselves or as complementary tools to quit smoking. The objective of the study was to determine the use of information and communication technologies (ICTs) in the smoking population attended in primary care, and describe the major factors associated with its use.

**Methods:**

Descriptive observational study in 84 health centres in Cataluña, Aragon and Salamanca. We included by simple random sampling 1725 primary healthcare smokers (any amount of tobacco) aged 18–85. Through personal interview professionals collected Socio-demographic data and variables related with tobacco consumption and ICTs use were collected through face to face interviews Factors associated with the use of ICTs were analyzed by logistic regression.

**Results:**

Users of at least one ICT were predominantly male, young (18–45 years), from most favoured social classes and of higher education. Compared with non-ICTs users, users declared lower consumption of tobacco, younger onset age, and lower nicotine dependence. The percentages of use of email, text messages and web pages were 65.3%, 74.0% and 71.5%, respectively. Factors associated with the use of ICTs were age, social class, educational level and nicotine dependence level. The factor most closely associated with the use of all three ICTs was age; mainly individuals aged 18–24.

**Conclusions:**

The use of ICTs to quit smoking is promising, with the technology of mobile phones having a broader potential. Younger and more educated subjects are good targets for ICTs interventions on smoking cessation.

## Background

Tobacco consumption is one of the leading preventable causes of death worldwide [1]; for instance, respiratory and cardiovascular diseases, and cancer are three well-established health effects of tobacco consumption among both smokers and non-smokers [2]. It has been estimated that in Spain smoking is the health problem that causes most mortality and morbidity. Consequently, it also originates higher health costs [3]. The percentage of daily smokers aged 15 or older in Spain was 24.0% (27.9% in men and 20.2% in women) according to the last national survey conducted in 2011–12 [4]. A large number of Spanish smokers declared their willingness to quit smoking (approximately 70%) and 27.4% have tried it on the past year [3], but merely 3–5% of them accomplished it [3, 5, 6].

Interventions to quit smoking are one of the most cost-effective methods to improve the health of the population [3, 5–8]. It is well accepted that the more intensive the intervention the best cessation rates; for instance whilst a 5% cessation per year is reached with minimum advice, a 20% can be achieved with more intensive interventions [9, 10]. Common interventions to help smokers quit are based on medical advice and pharmacological assistance as nicotine replacement therapy and bupropion. Alternative interventions such as hypnosis, acupuncture, exercise and opioid agonist have assisted some people in smoking cessation but there is not a clear consensus on its efficacy [8, 11].

The use of technologies that offers access to information via telecommunications (Information and communication technology (ICTs)), is augmenting progressively mainly internet, email and cell phone use; in fact, we live in a growingly electronic world [12–14]. For instance, worldwide use of mobile phones increased by 15.5% in 2010, reaching 78 telephone lines per 100 inhabitants, with a cumulative average growth between 2005 and 2010 of 19.5%. Likewise, Internet use had a 13% of growth in 2010, exceeding the number of 2044 millions of users. Europe and USA are the two geographical areas with the largest number of internet users: 67% and 50.7%, respectively [12]. According to The National Observatory for Telecommunications and the Information Society, in Spain 2011, 82.9% of people aged 15 or older had a mobile phone and 66.3% had accessed internet at least once [15]. If these data are analyzed as development indicators and, especially in the case of internet, as potential tools to change behaviours, these technologies can pose a great influence on health policies (directly or indirectly) [16, 17].

The use of ICTs has been growing in several fields including medicine; for instance, appointments can be scheduled on-line and analytical results or health information can be consulted on internet. ICTs technology has also been adopted on lifestyle interventions including smoking cessation [3, 8]. Recent reviews have analyzed the efficacy of web-based interventions on smoking cessation [18, 19] although results remain inconclusively [20]. Advantages of using ICTs on smoking cessation programs include its wide use, time and cost savings (they can diminish visits to the health centre and the possibility to check the information or messages/mails at patient or health professional convenience) and the possibility to supply personalized support [21].

Some recent systematic reviews that evaluates smoking cesation programs that use computer, internet, mobile phone and other electronic aids conclude their effectiveness, altough small and mainly at long term, on smoking cessation compared to no intervention or standard counseling [22–24].

Describing the use of ICTs among patients attending primary care could help us elucidate the viability of an ICT intervention in smoking cessation in primary care. For instance, our research group will compare: brief advice vs. personalized E-mail tracking (TABATIC study) [25]. Therefore, the aim of the present study is to determine the use of ICT in the smoking population attended in primary healthcare and to describe the main factors associated with its use.

## Methods

### Study design

We conducted a cross-sectional study to describe the use of ICTs among smokers attended in primary care as well as the main factors associated with that use. The study was multicentre; 195 healthcare professionals (general practitioners or nurses) of 84 primary healthcare centres of the Spanish public health system in Cataluña, Aragón and Salamanca (Spain) participated in the recruitment of patients.

### Subjects

Sample size was calculated according to the project’s aim, which was to estimate the use of ICTs in smokers attended in primary care. Assuming an alpha risk of 0.05 and a beta risk of 0.20 in a two-sided test and a no-response rate of 20%, 481 subjects were needed. We considered that at least half of the Spanish population in 2011 had access to internet and mobile phones [15]. From November 2011 to January 2012, individuals aged 18–75 who answered positively to the question “Do you smoke?” (independently of the amount) and signed the consent form were recruited by random sampling. Patients were recruited as they visited the primary care team and each day the first two subjects that fulfilled the inclusion criteria were invited to participate. We asked the health professionals to recruit participants at least two days per week. In case the patient declined to participate in the study, the health professional gathered age and sex and the reason of the refusal. Recruitment and data collection was performed by the health professional that commonly attends the patient.

People suffering from terminal illnesses, severe psychiatric disorders, addiction to other psychoactive substances, or who did not consent to participate in the study were excluded. Of the 1850 patients that fulfilled the inclusion criteria, 1725 agreed to participate (93.2%). The percentage of participation was similar between men and women in each age stratum (36–45 years old, p = 0.913; 46–65 years old, p = 0.176; >65 years old, p = 0.246), except that less men (93.1% vs. 98.0%, p = 0.008) accepted to participate among individuals aged 35 and younger.

The study protocol was reviewed and approved by the Health Care Ethics Committee and the Clinical Research Ethics Committee of the Primary Health Care University Research Institute-IDIAP Jordi Gol located in Barcelona, Spain.

### Study variables

The following information was obtained by healthcare professionals collected through face to face interviews: age, sex, educational level, occupational social class, civil status, ICTs (email, text messaging and web pages) availability and use, self-declared daily tobacco consumption in cigarettes per day, smoking onset age, number of previous attempts (of at least of 24 hours) to quit smoking, maximum abstinence time (in days), pharmacological treatment used on previous attempts (nicotine substitutes, Bupropion, Vareniclina), environmental exposure to smoke from family, workmates and friends and nicotine dependence level measured by the simplified two-question Fagerström test classified as low, medium and high [26]. Educational level refers to the maximum level of finalized studies, classified into: no formal studies, primary studies, secondary and university. Subsequently, it was recoded into lower than secondary and ≥ secondary.

To assign occupational social class we used the Spanish classification, which is based on Goldthorpe’s scheme which was designed to facilitate international comparisons [27]. Consequently, social class was assigned through the current or last occupation of the patient; in cases where the subject had not worked, through the current or last occupation of the head of the household [28]. The classification includes five well-established main social groups, but was subsequently collapsed into smaller number of categories: manual (social classes IV-V) and non-manual workers (the rest) to undertake analysis [27].

The information in the use of the three ICTs (E-mail, text messages and web pages) was gather by the following two questions: Do you use electronic mail (or internet/web page or sms)?. Possible answers were 'No’ or 'Yes’. If the participant responded yes then the interviewer asked for the frequency of use; possible answers were: 'less than once a week’’, once a week’ or 'more than once a week’. Consequently, the use of the three ICTs was grouped into four categories: 'no use’, 'less than once a week’, 'once a week’ and 'more than once a week’. Subsequently, it was recoded into 'no use’ and 'low frequency of use’ and 'mid/high frequency of use’.

This study included other variables that are not presented in this paper.

### Statistical analysis

Results are expressed as mean and standard deviation (SD) for quantitative variables or by frequency distribution for qualitative variables. Pearson’s Chi-square test for independence or homogeneity was applied to assess the relationship between two categorical variables. The Student’s *t*-test and ANOVA for independent samples were used to analyze associations between dichotomic and continuous normal qualitative variables, respectively. Mann-Whitney’s U and Kruskal-Wallis test were used to compare dichotomic and continuous variables if they did not follow a normal distribution. Binary logistic models were used to assess the associations between sociodemographic and tobacco consumption factors and ICTs use. The level of statistical significance was set at 0.05, and all tests were two-tailed. Statistical analyses were conducted using SPSS, version 17.0 (SPSS Inc, Chicago, IL).

## Results

A total of 1725 smokers participated in the study; mean age 45.5 years (SD: 13.6 years) and 865 (51.1%) were male. Characteristics of participants are shown in Table 1. Participants were more likely to be married (63.5%), manual workers (59.5) and 52.5% had completed, at least, secondary education. Mean age of starting tobacco consumption was 17.2 (SD: 4.5) and the mean number of self-declared cigarettes smoked per day was 15.4 (SD: 9.3). Half of the participants declared a low dependency on nicotine. 74.5% of participants declared previous attempts to quit smoking, and 76.6% of those did not use any medication; in cases where they had used medication, a nicotine substitute was the most frequently used. Patients included tended to live in a non-smoke-free environment; of those who had a partner, 47.9% declared living with a partner that smoked. Of those who were working or studying, 55.2% declared having co-workers that smoked; 65.4% of the participants declared that their friends lived in a smoking environment.

**Table 1:** **Comparison of main sociodemographic features and tobacco consumption variables among non users vs. users of any ICT**

	Total		Non users		Users		***P*** -value
	***N***	(%)	***N***	(%)	***N***	(%)	
Participants	1725		269	(15.6)	1456	(84.4)	
Gender	1725						<0.001
Male	865	(51.1)	180	(33.1)	685	(53.0)	
Female	860	(49.9)	89	(66.9)	771	(47.0)	
Age (years). Mean (SD).	45.54 (13.65)		55,60 (11.97)		41,39 (12.06)		<0.001
Age group	1725						<0.001
18-35	451	(26.1)	6	(2.2)	445	(30.6)	
36-45	402	(23.3)	21	(7.8)	381	(26.2)	
46-65	733	(42.5)	159	(59.1)	574	(39.4)	
>65	139	(8.1)	83	(30.9)	56	(3.8)	
Marital status	1725						<0.001
Married	1096	(63.5)	198	(73.6)	898	(61.7)	
Single	395	(22.9)	27	(10.0)	368	(25.3)	
Separate	174	(10.1)	18	(6.7)	156	(10.7)	
Widow/er	60	(3.5)	26	(9.7)	34	(2.3)	
Social class	1658						<0.001
Most favored: Non-manual	672	(40.5)	40	(16.5)	632	(44.7)	
Disadvantaged: Manual	986	(59.5)	203	(83.5)	783	(55.3)	
Educational level	1723						<0.001
Lower secondary education	819	(47.5)	216	(80.3)	603	(41.5)	
Secondary/higher education	904	(52.5)	53	(19.7)	851	(58.5)	
Number cigarettes/day. Mean (SD).	15.39 (9.28)		17.35 (11.40)		15.03 (8.80)		<0.001
Age of initiation consumption. Mean (SD). *Mean (SD)* MEAN	17.21 (4.55)		18.62 (6.64)		16.96 (4.01)		<0.001
Fagerström test. Mean (SD)	2.35 (1.63)		2.62 (1.74)		2.31 (1.61)		0.004
Fagerström test	1693						<0.001
Low	862	(50.9)	130	(48.9)	732	(51.3)	
Medium	691	(40.8)	98	(36.8)	593	(41.6)	
High	140	(8.3)	38	(14.3)	102	(7.1)	
Attempts at smoking cessation. Mean (SD).	2.2 (2.4)		2.1 (2.2)		2.2 (2.5)		0.480
Smoking environment of partner*	1448						<0.001
Yes	695	(47.9)	69	(31.7)	626	(50.9)	
No	753	(52.1)	149	(68.3)	604	(49.1)	
Presence of smoking in the home*	1673						0.001
Yes	939	(87.5)	121	(47.1)	818	(57.8)	
No	734	(12.5)	136	(52.9)	598	(42.2)	
Workplace/smoking studies*	1351						0.970
Yes	745	(55.2)	73	(55.3)	672	(55.1)	
No	606	(44.8)	59	(44.7)	547	(44.9)	
Smoking environment of friends	1725						0.570
Yes	1129	(65.4)	172	(63.9)	957	(65.7)	
No	596	(34.6)	97	(36.1)	499	(34.3)	
Have made some attempt to quit smoking	1582						0.830
Yes	1179	(74.5)	349	(74.9)	830	(74.4)	
No	403	(25.5)	117	(25.1)	286	(25.6)	
Pharmacoterapy used for smoking cessation^†^	1137						0.210
Nicotine replacement therapy	132	(11.6)	17	(9.7)	115	(12.0)	
Bupropion	27	(2.4)	5	(2.8)	22	(2.3)	
Varenicline	43	(3.8)	4	(2.3)	39	(4.1)	
≥2 treatments	64	(5.6)	5	(2.8)	59	(6.1)	
No medication	871	(76.6)	145	(82.4)	726	(75.5)	
Chronic medication intake	1137						0.049
Some medication	266	(23.4)	31	(17.6)	235	(24.5)	
No medication	871	(76.6)	145	(82.4)	726	(75.5)	

SD: Standard deviation.

*P*-value derived from the Chi-square test and ANOVA in categorical and continuous variables, respectively.

^*^Not taking into account cases of “not applicable”:without partner, living alone or not working or studying.

†In the last attempt to quit.

*P*-value derived from the Chi-square test and ANOVA in categorical and continuous variables, respectively.

When comparing non-users of any ICT (n = 269) with users of at least one ICT (any frequency of use), ICTs users (n = 1456) tended to be male, middle/young (18 to 45 years), non-manual workers and had a higher educational level (all p <0.001). The users of at least one ICT also reported lower consumption of tobacco, had started using tobacco at a younger age and a higher percentage of them had lower nicotine dependence, tended to live with partners who smoke and in not smoke-free homes and consumed chronic medication. No statistically significant differences were found neither regarding the number of previous attempts to quit smoking nor other smoking environments (Table 1).

Frequency of use of the three ICTs is specified in Table 2. Self-reported use of E-mail, text messaging and web pages were 65.3% (49.8% for high use), 74% (50.8% for high use) and 71.5% (56.0% for high use), respectively. Descriptive analysis showed that more high frequency users were women, middle/young (18 to 45) and individuals with a higher educational level. Those individuals from lower social classes tended to declare no use or lower use of E-mail, text messaging and internet. Regarding tobacco consumption variables, the number of cigarettes smoked per day and nicotine dependence was higher among non users of ICTs. Conversely, users, of both low and high frequency, declared a lower age of start of tobacco consumption.

**Table 2:** **Comparison of the frequency of use of three ICTs (email, sms and the web pages) according to sociodemographic variables and tobacco consumption of the participants in the study “Usage profile of ICTs”**

	Total		Don’t use E-mail		Low use of E-mail		Mid/high use of E-mail		***P*** -value	Don’t use web pages		Low use of webs pages		Mid/high use of web pages		***P*** -value
	***N***	(%)	***N***	(%)	***N***	(%)	***N***	(%)		***N***	(%)	***N***	(%)	***N***	(%)	
Participants	1725		599	(34.7)	267	(15.5)	859	(49.8)		492	(28.5)	267	(15.5)	966	(56.0)	
Gender (N = 1725)									<0.001							<0.001
Male	865	(50.1)	337	(56.3)	135	(50.6)	393	(45.8)		279	(56.7)	124	(46.4)	462	(47.8)	
Female	860	(49.9)	262	(43.7)	132	(49.4)	466	(54.2)		213	(43.3)	143	(53.6)	504	(52.2)	
Age (years) (N = 1725)	45.5 ± 13.6		54.4 ± 12.2		42.3 ± 12.2		40.3 ± 11.8		<0.001	56.0 ± 11.9		45.2 ± 11.9		40.3 ± 11.8		<0.001
Age group									<0.001							<0.001
18-35	451	(26.1)	42	(7.0)	87	(32.6)	322	(37.5)		25	(5.0)	57	(21.3)	369	(38.2)	
36-45	402	(23.3)	87	(14.5)	66	(24.7)	249	(29.0)		65	(13.2)	70	(26.2)	267	(27.6)	
46-65	733	(42.5)	355	(59.3)	107	(40.1)	271	(31.5)		293	(59.6)	132	(49.4)	308	(31.9)	
>65	139	(8.1)	115	(19.2)	7	(2.6)	17	(2.0)		109	(22.2)	8	(3.0)	22	(2.3)	
Social class (N = 1658)									<0.001							<0.001
Most favored/NM	672	(40.5)	101	(18.1)	78	(29.9)	493	(58.8)		81	(17.7)	80	(31.1)	511	(54.2)	
Disadventaged-M	986	(59.5)	458	(81.9)	183	(70.1)	345	(41.2)		377	(82.3)	177	(68.9)	432	(45.8)	
Educational level (N = 1723)									<0.001							<0.001
<Secondary	819	(47.5)	439	(51.7)	138	(51.7)	242	(28.2)		381	(77.4)	126	(47.2)	312	(32.4)	
≥Secondary/Higher	904	(52.5)	129	(48.3)	129	(48.3)	615	(71.8)		111	(22.6)	141	(52.8)	652	(67.6)	
Num. Cigarettes/ day	15.4 ± 9.3		16.9 ± 10.4		15.3 ± 8.7		14.4 ± 8.4		<0.001	17.2 ± 10.9		14.7 ± 8.1		14.7 ± 8.6		<0.001
Age of initiation consumption	17.2 ± 4.5		18.0 ± 6.0		16.5 ± 3.2		16.9 ± 3.6		<0.001	18.3 ± 6.3		16.9 ± 3.9		16.8 ± 3.5		<0.001
Fagerström test	2.4 ± 1.6		2.6 ± 1.7		2.4 ± 1.6		2.1 ± 1.6		<0.001	2.6 ± 1.7		2.4 ± 1.5		2.2 ± 1.6		<0.001
Attempts smoking cessation	2.2 ± 2.4		2.1 ± 2.3		2.2 ± 2.4		2.3 ± 2.5		0.182	2.1 ± 2.3		2.1 ± 2.4		2.3 ± 2.5		0.185
	**Total**				**Don’t use sms**				**Low use of sms**			**Mid/high use of sms**				***P*** **-value**
	***N***		**(%)**		***N***		**(%)**		***N***	**(%)**		***N***		**(%)**		
					448		(26.0)		400	(23.2)		877		(50.8)		
Gender (N = 1725)																<0.001
Male	865		(50.1)		305		(68.1)		199	(49.8)		361		(41.2)		
Female	860		(49.9)		143		(31.9)		201	(50.3)		516		(58.8)		
Age (years) (N = 1725)	45.5 ± 13.6				54.4 ± 12.3				47.3 ± 11.9			39.7 ± 11.8				<0.001
Age group																<0.001
18-35	451		(26.1)		32		(7.1)		66	(16.5)		353		(40.3)		
36-45	402		(23.3)		52		(11.6)		100	(25.0)		250		(28.5)		
46-65	733		(42.5)		272		(60.7)		209	(52.3)		252		(28.7)		
>65	139		(8.1)		92		(20.5)		25	(6.3)		22		(2.5)		
Social class (N = 1658)																<0.001
Most favored/NM	672		(40.5)		102		(24.3)		142	(36.8)		428		(50.2)		
Disadventaged-M	986		(59.5)		317		(75.7)		244	(63.2)		425		(49.8)		
Educational level (N = 1723)																<0.001
<Secondary	819		(47.5)		302		(67.4)		205	(51.3)		312		(35.7)		
≥Secondary/Higher	904		(52.5)		146		(32.6)		195	(48.8)		563		(64.3)		
Num. Cigarettes/ day	15.4 ± 9.3				16.7 ± 10.9				16.0 ± 9.3			14.4 ± 8.3				<0.001
Age of initiation consumption	17.2 ± 4.5				17.9 ± 5.7				17.3 ± 4.8			16.8 ± 3.7				<0.001
Fagerström test	2.4 ± 1.6				2.5 ± 1.7				2.4 ± 1.6			2.2 ± 1.6				0.003
Attempts smoking cessation	2.2 ± 2.4				2.2 ± 2.4				2.1 ± 2.2			2.3 ± 2.5				0.755

ICT: Information and Communication Technologies.

Low use: ≤1 time per week; Mid/high use: >1 time per week.

*p*-value derived from the Chi square test and ANOVA in categorical and continuous variables respectively.

Low use: ≤1 time per week; Mid/high use: >1 time per week.

*p*-value derived from the Chi square test and ANOVA in categorical and continuous variables respectively.

Finally, we analyzed factors affecting ICTs use (Tables 3, 4 and 5) by comparing no use vs. low frequency of use (OR1) and no use vs. mid/high frequency of use (OR2). Binary logistic adjusted analysis showed that age was the strongest predictor of E-mail frequency use (for individuals aged 18–35, OR1 = 33.4; CI95%:13.97-80.25 and OR2 = 60.0; CI95%: 30.1-95.3). Higher social class (OR1 = 1.61; CI95%:1.08-2.34 and OR2 = 4.29; CI95%: 3.11-5.93) and educational level (OR1 = 2.22; CI95%: 1.56-3.15 and OR2 = 4.08; CI95%: 3.03-5.48) were also positively associated to the frequency of use of E-mail. Low dependence to nicotine was associated with a mid/high use of E-mail use (OR2 = 2.03; CI95%: 1.22-3.38). Further adjustments did not materially alter these associations. These results are consistent with crude analysis.

**Table 3:** **Main predictors of the use of E-mail**

	Use of E-mail					
	***Low frequency of use***			***Mid/high frequency of use***		
	OR1	(95% CI)	***P*** -value	OR2	(95% CI)	***P*** -value
**CRUDE OR**						
**Gender**						
Male	1.00		<0.001	1.00		0.169
Female	0.65	(0.53-0.81)		0.822	(0.62-1.09)	
**Social class**						
Disadventaged-M	1.00		<0.001	1.00		<0.001
Most favored_NM	1.93	(1.37-2.72)		6.48	(5.02-8.37)	
**Educational level**						
<Secondary	1.00		<0.001	1.00		<0.001
≥Secondary	2.56	(1.89-3.46)		6.97	(5.52-8.81)	
**Age Group**						
>65	1.00			1.00		
46-65	4.95	(2.24-10.94)	<0.001	5.16	(3.03-8.80)	<0.001
36-45	12.4	(5.45-28.51)	<0.001	19.36	(11.07-34.06)	<0.001
18-35	34.0	(14.59-79.40)	<0.001	51.86	(28.39-94.71)	<0.001
**Fagerström test**						
High	1.00			1.00		
Medium	1.46	(0.87-2.46)	0.152	2.33	(1.55-3.51)	<0.001
Low	1.47	(0.88-2.45)	0.144	2.74	(1.83-4.09)	<0.001
ADJUSTED OR*						
**Gender**						
Male	1.00		0.613	1.00		0.300
Female	0.926	(0.66-1.27)		0.86	(0.65-1.14)	
**Social class**						
Disadventaged-M	1.00		0.019	1.00		<0.001
Most favored_NM	1.61	(1.08-2.34)		4.293	(3.11-5.93)	
**Educational level**						
<Secondary	1.00		<0.001	1.00		<0.001
≥Secondary	2.22	(1.56-3.15)		4.08	(3.03-5.48)	
**Age Group**						
>65	1.00			1.00		
46-65	4.64	(2.06-10.45)	<0.001	5.46	(2.97-10.04)	<0.001
36-45	11.9	(5.10-28.11)	<0.001	21.9	(11.4-41.9)	<0.001
18-35	33.4	(13.97-80.25)	<0.001	60.0	(30.1-95.3)	<0.001
**Fagerström test**						
High	1.00			1.00		
Medium	1.25	(0.71-2.19)	0.436	1.92	(1.15-3.20)	0.013
Low	1.24	(0.71-2.17)	0.448	2.03	(1.22-3.38)	0.006

M = Manual; NM = Non Manual.

Low frequency of use: ≤1 time per week; Mid/high frequency of use: >1 time per week.

OR: Odd Ratio; OR = 1 denotes reference category.

OR1: Odds Ratio of low frequency use vs. no use.

OR2: Odds Ratio of mid/high frequency use vs. no use.

Adjusted OR*: OR adjusted for potential confounders: in the case of gender by age, social class and level of education. Fagerström test was adjusted for all other variables.

*P*-value derived from the Wald test.

**Table 4:** **Main predictors of the use of SMS**

	Use sms					
	***Low frequency of use***			***Mid/high frequency of use***		
	OR1	(95% CI)	***P*** -value	OR2	(95% CI)	***P*** -value
**CRUDE OR**						
**Gender**						
Male	1.00		<0.001	1.00		<0.001
Female	2.15	(1.63-2.85)		3.0522	(2.40-3.88)	
**Social class**						
Disadventaged-M	1.00		<0.001	1.00		<0.001
Most favored_NM	1.81	(1.33-2.45)		3.13	(2.41-4.06)	
**Educational level**						
<Secondary	1.00		<0.001	1.00		<0.001
≥Secondary	1.972	(1.49-2.60)		3.73	(2.93-4.75)	
**Age Group**						
>65	1.00			1.00		
46-65	2.82	(1.75-4.56)	<0.001	3.87	(2.36-6.36)	<0.001
36-45	7.08	(4.06-12.32)	<0.001	20.10	(11.57-34.95)	<0.001
18-35	7.59	(4.12-13.98)	<0.001	46.13	(25.59-83.16)	<0.001
**Fagerström test**						
High	1.00			1.00		
Medium	1.62	(1.01-2.63)	0.177	2.19	(1.44-3.35)	<0.001
Low	1.38	(0.86-2.21)	0.047	2.18	(1.44-3.30)	<0.001
ADJUSTED OR*						
**Gender**						
Male	1.00		<0.001	1.00		<0.001
Female	1.8926	(1.40-2.57)		2.5186	(1.88-3.34)	
**Social class**						
Disadventaged-M	1.00		0.122	1.00		0.001
Most favored_NM	1.33	(0.93-1.91)		1.79	(1.28-2.50)	
**Educational level**						
<Secondary	1.00		0.017	1.00		<0.001
≥Secondary	1.502	(1.07-2.10)		2.31	(1.69-3.16)	
**Age Group**						
>65	1.00			1.00		
46-65	2.65	(1.56-4.49)	<0.001	3.11	(1.79-5.37)	<0.001
36-45	7.14	(3.88-13.13)	<0.001	16.2	(8.76-29.80)	<0.001
18-35	7.04	(3.65-13.56)	<0.001	33.8	(17.87-64.12)	<0.001
**Fagerström test**						
High	1.00			1.00		
Medium	1.41	(0.84-2.36)	0.432	1.29	(0.79-2.13)	0.307
Low	1.28	(0.73-2.05)	0.196	1.473	(0.89-2.43)	0.132

M = Manual; NM = Non Manual.

Low frequency of use: ≤1 time per week; Mid/high frequency of use: >1 time per week.

OR: Odd Ratio; OR = 1 denotes reference category.

OR1: Odds Ratio of low frequency use vs. no use.

OR2: Odds Ratio of mid/high frequency use vs. no use.

Adjusted OR*: OR adjusted for potential confounders: in the case of gender by age, social class and level of education. Fagerström test was adjusted for all other variables.

*P*-value derived from the Wald test.

**Table 5:** **Main predictors of the use of web pages**

	Use of web pages					
	***Low frequency of use***			***Mid/high frequency of use***		
	OR1	(95% CI)	***P*** -value	OR2	(95% CI)	***P*** -value
**CRUDE OR**						
**Gender**						
Male	1.00		0.007	1.00		0.001
Female	1.51	(1.12-2.04)		1.432	(1.15-1.78)	
**Social class**						
Disadventaged-M	1.00		<0.001	1.00		<0.001
Most favored_NM	2.10	(1.47-3.00)		5.50	(4.19-7.23)	
**Educational level**						
<Secondary	1.00		<0.001	1.00		<0.001
≥Secondary	3.84	(2.79-5.29)		7.17	(5.58-9.22)	
**Age Group**						
>65	1.00			1.00		
46-65	6.145	(2.90-12.95)	<0.001	5.21	(3.21-8.46)	<0.001
36-45	14.67	(6.64-32.44)	<0.001	20.3	(11.95-34.65)	<0.001
18-35	31.0	(13.17-73.28)	<0.001	73.1	(28.39-94.71)	<0.001
**Fagerström test**						
High	1.00			1.00		
Medium	2.196	(1.22-3.92)	0.009	2.25	(1.53-3.31)	<0.001
Low	2.02	(1.13-3.61)	0.017	1.974	(1.33-2.93)	0.001
ADJUSTED OR*						
**Gender**						
Male	1.00		0.990	1.00		0.048
Female	1.01	(0.71-1.41)		0.74	(0.55-0.99)	
**Social class**						
Disadventaged-M	1.00		0.148	1.00		<0.001
Most favored_NM	1.36	(0.90-2.07)		3.42	(2.42-4.84)	
**Educational level**						
<Secondary	1.00		<0.001	1.00		<0.001
≥Secondary	3.42	(2.36-4.97)		4.51	(3.29-6.20)	
**Age Group**						
>65	1.00			1.00		
46-65	5.47	(2.52-11.88)	<0.001	5.54	(3.16-9.72)	<0.001
36-45	12.8	(5.6-29.4)	<0.001	23.5	(12.6-43.7)	<0.001
18-35	27.0	(11.0-66.2)	<0.001	84,4	(42.2-169.2)	<0.001
**Fagerström test**						
High	1.00			1.00		
Medium	1.873	(0.99-3.51)	0.051	1.51	(0.96-2.51)	0.083
Low	1.76	(0.94-3.30)	0.077	1.42	(0.87-2.32)	0.161

M = Manual; NM = Non Manual.

Low frequency of use: ≤1 time per week; Mid/high frequency of use: >1 time per week.

OR: Odd Ratio; OR = 1 denotes reference category.

OR1: Odds Ratio of low frequency use vs. no use.

OR2: Odds Ratio of mid/high frequency use vs. no use.

Adjusted OR*: OR adjusted for potential confounders: in the case of gender by age, social class and level of education. Fagerström test was adjusted for all other variables.

*P*-value derived from the Wald test.

All these factors were also associated to SMS and internet frequency of use, except that dependence to nicotine did not remain statistically significant. Conversely, women were more likely to use SMS (OR1 = 2.15; CI95%: 1.63-2.85 and OR2 = 3.05; CI95%: 2.40-3.88).

When the frequency of E-mail, SMS and internet use was analyzed separately by age, social class and educational level the influence of the other factors was similar as among the whole sample studied (results not shown); for instance, the direction of the associations of young age and higher social class remained analogous and statistically significant in both, individuals with high and low educational level.

## Discussion

### Principal findings

By means of a representative sample of the smoking population attended in the public primary care system we have studied the differences among smokers in relation to the use of different ICTs (Internet, Email and SMS messages), including among the variables demographic characteristics, socioeconomic status and smoking profile. This study shows that the three studied ICTs are widely used, especially among the population under 45, women, more favoured social class, of higher education level and those with lower consumption. It also showed that cigarette consumption had started at a younger age in ICTs users.

Our data show that 84.4% of smokers use some of the ICTs studied; being the use of Internet, sms and E-mail and 71.5% (56.0% for high use), 74.0% (50.8% for high use) and 65.3% (49.8% for high use) respectively. The frequency of access to web of our study is comparable to the general population of Spain in 2012 (70.8%) [15] and to some international studies (65.5%) [14, 29]. Among smokers, Hunt et al. showed that 63.5% were internet users [30]. Some studies have attempted to characterize the group of internet users (not only smokers) who are interested in finding information about smoking on health-related web pages, but not a clear common profile has been found [31, 32]. Weaver and colleagues suggested that females, white respondents, people aged 55–64 and computer owners were positively associated with the interest of finding information on the web [32]. Our study shows that smokers from higher social class and educational level, as well as young age are associated to web use which is in accordance with some other studies [30, 33, 34]. Regarding gender, our study shows more women users (52.2-53.6%) along with other studies [33, 35], but results remain inconclusively [30, 34]. Chander and collaborators found that higher education was associated with the use of these ICTs among HIV carriers [36].

There are few studies that report sms use among the general population. According to the Forrester Research Mobile Media Application Spending Forecast, 'more than 6 billion of SMS are sent each day and text message users receive an average of 35 messages per day’. Concretely, more than 80% of the US population owning a mobile phone and with almost 70% of these phone owners regularly sending or receiving text messages [37, 38]. In Spain, there are 33.4 millions of cell phone users (85.8% of people aged 15 or older) [15]. No data has been found regarding sms use among smokers, except for the study published by Chander et al. that reported that 39% of HIV positive smokers used sms and its use was mainly associated to higher educational level [36]. Data from the control group of a clinical trial that used text message to help smokers to quit showed that mostly were unemployed, students and manual workers (55%); which was similar to our findings (63.2-49.8%), but were younger and tended to be more males (55% vs. 43.9%) [39].

Regarding the use of email, we have not found data to inform the use of these ICTs by smokers. Data concerning the populations that have participated in trials using email in treating smokers show similarities in some socio-demographic factors (younger age and predominantly female participants) [40, 41], although Polosa and collegues reported a higher participation of men [42].

Data from our study showed more consumption of tobacco among non-users of the three technologies (mean cigarettes per day: 17.3 (SD: 11.4)) compared to users (mean cigarettes per day: 15.0 (SD: 8.80)). Stoddard & Auguston reported similar cigarette consumption but did not find differences between those who used internet and those who didn’t [34]. Besides, our data showed that age of onset of smoking we report that our study shows differences between among ICTs users was lower (17.0 (SD: 4.0)) than non users (18.6 (SD: 6.6)). Neither similar findings nor possible explanations have found in the literature.

The three technologies used show a very high use among smokers, which suggests that they could be potentially very useful in interventions based on exclusive use, use in combination or supporting face-to-face interventions. Each of the three technologies studied have their own advantages and disadvantages. Disadvantages include the development of a private and secure environment to regulate its use, refusal to use them and lack of experience and time on its use [21, 34, 43, 44]. E-mail and SMS would probably be the most feasible to use since both patient and sanitary professional can check it at their own convenience which allows certain time to respond (preferably in the first 48 hours), can diminish visits to the primary care center, are helpful on sending reminders, improve medication adherence and self-management of some chronic illnesses and can provide visual information [21, 22, 25]. Whilst, E-mail is a quite cheap technology, in Spain, SMS comprise higher costs in Spain since they are not chargeless. Although websites share some of these advantages can transmit a feeling of impersonality to certain users. Additionally, the use of these technologies should be tailored; thus, the potential therapeutic use rises if these technologies are used by the sanitary professional who knows and treats the patient [18]. Moreover, the relationship among patient and sanitary professional can be deepened and encouraging attitudes can be generated if the experience is positive [43].

### Limitation and future directions

One of the main limitations of the study is its design; a cross-sectional study does not allow causal associations. This study was conducted in a population of smokers, and we were not able to acknowledge the differences between this group and the general population. In fact, participation in the study was offered to several referees of primary care research of all the Spanish territory and only those form Catalonia, Aragón and Salamanca accepted to participate; maybe the ICT use in other regions of Spain could be different. We only studied those who came to the primary healthcare centres, considering that the vast majority of the Spanish population attend them once a year [8, 45, 46] and were potential users of these technologies. Consequently, primary care can be an ideal setting to recruit participants from the general population to whom ICT based interventions can be tested.

We did not evaluate barriers to the use of these technologies and did not consider the costs associated with mobile phone use and texting, which may limit its use in behavioural interventions. However, it is an ongoing qualitative and quantitative research by our research group that will analyze the barriers and aids to the use of ICTs among smokers and health professionals. The qualitative study will also try to assess if patients would engage into a cessation program since we were not able to gather this information on the present study.

Regarding cell phone use, we have only asked for the use of sms in our population, but mobile applications (mobile Apps) are being developed to help smokers to quit and can post a new paradigm on smoking cessation [47]. No data was gathered among the use of social networking sites, such as Twitter, that can be a potential tool to support smoking cessation [48].

Our results show differences on nicotine dependence levels among ICT users and not users (p = 0.004); however the clinical relevance of this difference remains inconclusively in using ICTs to help smokers quit. Possibly, more intensive ICTs interventions (such as more sms or E-mails) will be needed on those smokers with higher nicotine dependence (with higher Fagerström test levels and higher number of cigarettes smoked).

The final model used in the binary logistic analysis includes social class and educational level that may result in over adjustment. However, in the context of a global economic crisis in our country, nowadays education cannot be used as an indicator of occupation since many people with higher education are currently working in jobs that do not match their educational level.

## Conclusions

In conclusion, the use of ICTs for smoking cessation is promising, since can reach an extensive range of population, with mobile phones being the technology with broadest potential. Considering the high prevalence of smoking in the general population and the broad use of ICTs in smokers, the health benefits are clear in terms of effectiveness and cost-effectiveness for treating these patients. By knowing the profile of smokers who use ICTs, primary care health professional can offer the possibility to use a specific ICT according to the smoker’s profile in order to maximise the probability of success. It is necessary to develop future clinical trials to determine the feasibility, acceptability and effectiveness of these technologies, as individual or complementary interventions with pharmacotherapy.

## Abbreviations

ICT: Information and communication technologies.
